# Enhancement of the heat conduction performance of boron nitride/cellulosic fibre insulating composites

**DOI:** 10.1371/journal.pone.0200842

**Published:** 2018-07-19

**Authors:** Zhihuai Yu, Xiu Wang, Huiyang Bian, Liang Jiao, Weibing Wu, Hongqi Dai

**Affiliations:** Jiangsu Co-Innovation Center for Efficient Processing and Utilization of Forest Resources, Nanjing Forestry University, Nanjing, China; Institute of Materials Science, GERMANY

## Abstract

The continuous development of high electrical equipment towards high power output requires better heat dissipation performance of internal insulation structure. It challenges the traditional paper-based insulating materials, with poor thermal conductivity. Introducing thermally conductive and electrically insulating filler into cellulose-based insulating material can enhance heat conduction performance. This work provided a method to prepare thermally conductive and electrically insulating BN/cellulosic fibre composites. And the thermal conductivity of the composites was remarkably increased via grafting APTES and adding dual-sized fillers. The thermal conductivity of the composite reached 0.682 W/(m•k) that increased by 387% with h-BN loading of 41.08 wt%. Simultaneously, BN fillers improved the insulating properties of the resultant composites. The dielectric constant, breaking strength of and volume resistivity of the composites reached 4.75, 9.2 kV/mm^-1^ and 4.72×10^14^ Ω•m, respectively. The resultant insulating material which has better heat conduction property may have a vast potential for future development in electrical equipment.

## Introduction

Cellulose is the most abundant nature polymer in the world [1,2]. Cellulose-based insulating materials are widely used in electrical equipment including generators, transformers, cables, etc [3,4]. With the advancement of electrical power technology, electrical equipment has been developing towards high power output and miniaturization. High voltage and high power operating electrical equipment will generate lots of heat [5]. However, the poor thermal conductivity of cellulose-based insulating materials will hinder heat dissipation. When the undesirable heat cannot be transferred out of the working electrical equipment timely and effectively, the sharply risen temperature will result in not only reduction of the working efficiency, but also the heat degradation of the internal insulating material or other horrible consequences [6,7]. Previous studies mainly concentrate on resin composites, the flexible thin cellulose-based thermal conductive material was rarely reported. Thus, developing cellulosic fibre composites with better heat conduction property has an impact on the existing cellulose-based insulating materials [8].

Introducing thermal conducting particles as filler into cellulose-based insulating material is a feasible method to improve its heat conduction performance [9]. The thermal conducting fillers mainly include metals (silver [10], copper, aluminum, etc), carbonic materials (graphite [11,12], carbon nanotube [13,14]) and inorganic ceramic materials (boron nitride [15,16], aluminum nitride, aluminium oxide, etc). Although metals or carbonic materials exhibit ultrahigh thermal conductivity, large amounts of free electrons within these materials inevitably lead to electrical conduction [17]. However, inorganic ceramic materials have desirable high thermal conductivity and excellent electrical insulation property [18]. Hexagonal boron nitride (h-BN) is a typical III-V compound, which has a similar crystal structure with graphite. This two-dimensional stratiform crystal structure provides h-BN intrinsic high thermal conductivity [19,20]. h-BN have the highest thermal conductivity among the ceramic materials. Its in-plane (001) thermal conductivity reaches 180~200 W/(m•k).And h-BN can maintain original shape at high temperature because of its extremely low thermal expansion coefficient. In the meantime, h-BN owns a high energy gap of 5.9 eV, and exhibits outstanding electrically insulation properties [18,19]. Based on these merits, h-BN is considered as a promising candidate of thermally conductive and electrically insulating filler.

Herein, the h-BN filler was mixed with fibre suspension to prepare BN/cellulosic fibre composites with higher thermal conductivity. To further enhance the heat conduction performance of the composites. The 3-aminopropyltr-iethoxysilane(APTES) was added as the modifier to improve the compatibility between BN fillers and cellulosic fibres. And the BN fillers with two different sizes were used to increase contact area between filler particles. In this case, a higher thermal conductivity of 0.682 W/(m•k) was achieved at BN loading of 41.08 wt%, which corresponds to a thermal conductivity enhancement of about 387% compared with traditional insulating paper. The resultant composites thereby exhibited higher thermal conductivity and better electrically insulation properties.

## Materials and methods

Unbleached softwood kraft pulp (USKP) was supplied from Bratsk, Ilim Pulp Co.; hexagonal boron nitride (h-BN) powders with size of 1~2 and 5 μm were purchased from Aladdin reagent Co.; 3-aminopropyltr-iethoxysilane (APTES) was supplied from Sinopharm Chemical reagent Co,; cationic polyacrylarmide (CPAM) was supplied from Eka Chemicals Co.; sodium hydroxide (NaOH), hydrochloric acid (HCl), ethanol and toluene were supplied from Nanjing Chemical reagent Co.

### h-BN surface modification

**Fig 1:**
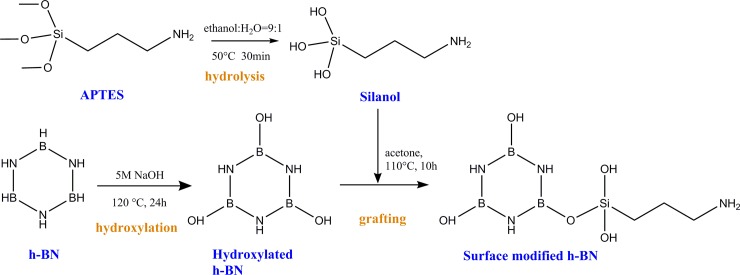
The preparation of surface modified h-BN particles.

The flow diagram of the surface modification of h-BN was shown in Fig 1. A certain amount of h-BN was hydroxylated in 5 mol/L NaOH solution at 120°C for 24 h to attach more hydroxyl groups onto the surface. A certain amount of APTES was dissolved into ethanol solution at 50°C for 30 min for pre-hydrolysis. Then hydroxylated h-BN and pre-hydrolyzed APTES solution were mixed in 120 ml absolute toluene to react at 110°C for 10 h. The surface modified h-BN was obtained.

### Preparation of BN/cellulosic fibre composites

The USKP was beaten to 55°SR using a ZQS2-23 beater (Shanxi University of Science & Technology, Xian, China). Then the BN fillers were mixed with USKP and stirred by a disintegrator (Frank-PTI, Austria). After 10000 revolutions, the suspension was flocculated by adding a small amount of CPAM. The suspension was poured into RK-2A sheet former (Frank-PTI, Austria) to prepare BN/cellulosic fibre composites. To ensure each sample has an accordant density, the resultant composites were pressed by a squeezer (Frank-PTI, Austria) at 0.5 MPa for 20 min. The resultant composite possessed a density of 0.8 g/cm^3^, a diameter of 20 cm and a grammage of 80 g/cm^2^.

### Characterization

The IRPrestige-21 FTIR spectrometer (Shimadzu Company, Japan) was used to identify the chemical structure of pristine h-BN and surface modified h-BN, capturing spectra between 500 and 4000 cm^-1^, at a resolution of 0.5 cm^-1^. A thermogravimetric analyzer (Q5000IR, TA instruments, USA) was applied to measure the thermal stability of the pristine h-BN and surface modified h-BN, the samples of approximately 10 mg were heated from 30°C to 800°C at a heating rate of 10°C∙min^-1^ under pure nitrogen with a flow rate of 40 ml∙min^-1^。The DZDR-S Transient surface heat source thermal conductivity instrument (Dazhan institute of electromechanical technology, Nanjing, China) was used to measure the thermal conductivity in a testing condition of 0.45w power for 160s at 25°C and 50% humidity. The breakdown strength of the samples was determined using a voltage breakdown tester (BDJC Beiguang precise instrument Co., Beijing, China), with a 50Hz sinusoidal AC power supply, at a voltage rise rate of 1 kV•min^-1^. The volume resistance was tested by an insulation resistance tester (ZC36, Shanghai Jingke Industrial Co., China) at 25°C and 50% humidity. The dielectric constant tester (GCSTD-A, Guance instrument Co., Beijing, China) was employed to measure the dielectric constant and dielectric loss angle. The surface morphology of the BN/cellulosic fibre composite was characterized using an environmental scanning electron microscope (Quanta-200, FEI, USA).

## Results and discussion

### h-BN surface modification

**Fig 2:**
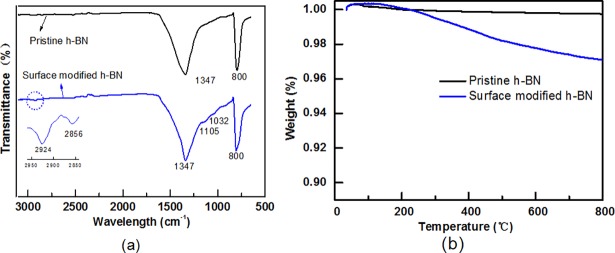
(a)FTIR spectra and (b)TGA curves of pristine h-BN and surface modified h-BN.

Fig 2(A) shows the infrared spectra of pristine h-BN and surface modified h-BN. The FT-IR spectrum of pristine h-BN displayed two strong absorption peaks at approximately 1347 and 800 cm^-1^, which were assigned to B-N out-of-plane stretching vibration band and B-N hexagonal ring bending vibration band, respectively [21]. Compared with pristine h-BN, new weak absorption peaks were observed at 2924 and 2856 cm^-1^ in surface modified h-BN. These bands corresponded to the anti-symmetric and symmetric vibration of -CH_2_- chains [22]. Moreover, new absorption peaks at 1032 and 1105 cm^-1^ were assigned to Si-O-C and Si-O-Si stretching vibration [22], indicating that the APTES was successfully grafted onto the surface of h-BN. As shown in Fig 2(B), the thermogravimetric curve of the pristine h-BN kept at a level up to 800°C. However, around 2.98 wt% weight loss was obtained for surface modified h-BN up to the temperature of 800°C due to the thermal degradation of the grafted groups.

### Heat conduction property

**Fig 3:**
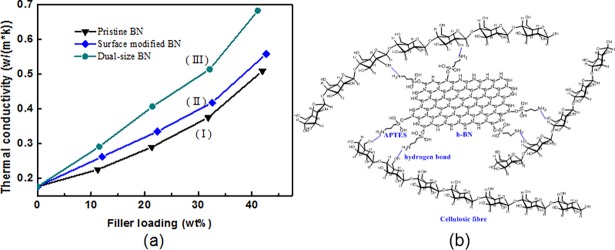
(a) The thermal conductivity of the composites with different BN fillers (b) Schematic diagram of the hydrogen bond between fillers and cellulosic fibres.

**Fig 4:**
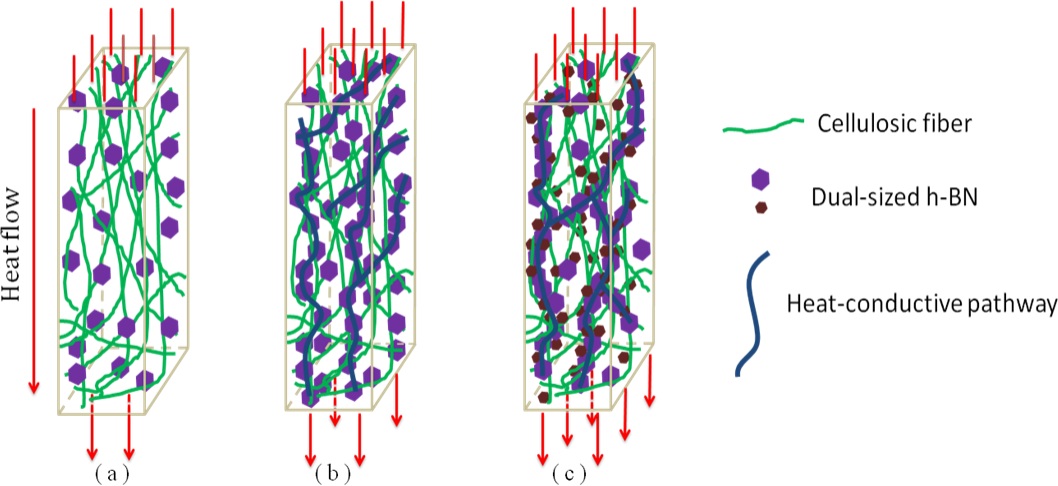
Schematic diagram of the filler distribution within the BN/cellulosic fibre composite (a) No heat-conductive pathway at low filler loading (b) Heat-conductive pathways gradually formed at high filler loading (c) More compact and integrated heat-conductive pathways with dual-size filler.

Fig 3(A) shows the thermal conductivity of the BN/cellulosic fibre composites with diverse filler particles (Table 1). It can be noted from the curves that thermal conductivity of all samples were obviously improved with the increasing content of h-BN filler concentration. Due to the disordered arrangement and porous structure, pure cellulosic fibre sample performed barely satisfactory in heat transport with a poor thermal conductivity of about 0.176 W/(m•k). The BN/cellulosic fibre composite possessed a relatively high thermal conductivity of 0.682 W/(m•k), which enhanced by 3.87 times compared with the sample without adding h-BN filler, indicating h-BN filler effectively improved the heat transfer property of the composite. Moreover, when the filler loading reached to 30 wt%, the thermal conductivity curves ascended in a more violent trend. This is because that at a low filler loading, most of the filler particles were dispersed in the interstices between cellulosic fibres [23], so that the sufficient contact cannot be built between the filler particles. In that case (shown in Fig 4(A)), the distribution of filler particles contributed slightly to the heat transport property of the BN/cellulosic fibre composites. Nevertheless, when the filler loading reached a certain critical value, which was called "permeation threshold" [24], the previous scattered filler particles gradually contacted each other to form a chain-liked or network-liked structure (shown in Fig 4(B)). The phonon was the dominating carrier in the process of heat conduction within solid materials. These chains or networks composed of filler particles were the desired channel of the phonon flow which named "heat-conductive pathway". Within the heat-conductive pathway, the interface scattering can be effectively avoided while the phonon flow delivered among the filler particles. And the phonon mean free path was lengthened. Hence, the interior calefaction caused by the collision of phonon can be reduced. The high-energy phonons were transferred to the low temperature region from the high temperature region along the orientation of heat-conductive pathways[18]. These pathways can considerably reduce the interior thermal resistance and improve the heat transport property of the material. Thus, the heat-conductive pathways were the main architecture which undertakes heat conduction within the particles-filled thermally conductive composite.

**Table 1:** Characteristics of h-BN fillers in different samples.

Curves	Surface modification	Size (μm)	Ratio (%)
Ⅰ	No	5	-
Ⅱ	Yes	5	-
Ⅲ	Yes	1~2 and 5	50 / 50

Moreover, compared with pristine h-BN fillers, grafting APTES onto the surface of fillers made further enhancement of the thermal conductivity of the BN/cellulosic fibre composites. Due to the presence of the APTES, there was a certain amount of polar amino groups on the surface of the h-BN particles, which provided a better compatibility to the interface between the h-BN particles and cellulosic fibres which also were abundant in polar hydroxyl groups on the surface [25,26]. In drying process, the hydrogen bonding formed between the amino groups and hydroxyl groups. In this case, the connection of the h-BN particles and cellulosic fibres was enhanced to effectively reduce the interface thermal resistance (Fig 3(B)) [27]. When the filler loading was 10, 20, 30 and 40wt%, the thermal conductivity of the composites added surface modified h-BN increased by 13.20%, 12.52%, 9.78% and 8.15%, respectively, compared with the composites which contained pristine h-BN. It can be observed that grafting APTRS onto the surface of h-BN had less effect on improving the thermal conductivity when the filler loading was relatively high. The main role of surface modified h-BN was to reduce the interface thermal resistance between fillers and cellulosic fibres [28]. As the filler loading reached a higher value, filler particles gradually formed chain-like structure, and the heat flow primary directly transferred along the filler-formed pathways. Then less heat flow passed through the interface between fillers and cellulosic fibres. In spite of the surface modification could enhance the mutual contact of fillers in some sense, the increase rate of the thermal conductivity decreased with the increasing of filler loading. It can seen in Fig 3(A), the thermal conductivity of the BN/cellulosic fibre composites that contained dual-sized fillers were much higher than the ones contain single-sized fillers. The advantage of dual-sized fillers addition was that the large size (about 5 μm) filler particles can constitute the skeleton of the heat-conductive pathways. Nonetheless, some voids might inevitably exist between filler particles due to the steric hindrance. When the phonons propagated, the phonon flow would occur interface scattering partly and result in reducing of heat transport efficiency. Using small size (about 1 μm) filler particles to plug these voids can form more compact and integrated heat-conductive pathways (shown in Fig 4(C)) to lengthen the phonon mean free path [29]. When the filler loading was 10, 20, 30 and 40wt%, the thermal conductivity of the BN/cellulosic fibre composite that contains dual-sized fillers increased by 11.70%, 17.65%, 24.61% and 26.73% respectively, which were much higher than the ones contain single-sized fillers. On the contrary of surface modification, the dual-sized fillers addition made better effects on the thermal conductivity of the composites as the filler loading increased. As mentioned above, the higher filler loading, the more significant part the heat-conductive pathways took for enhancement of the heat conductive performance of the composites [30]. And dual-sized fillers addition was just to enhance the propagation capacity of phonon flow within the heat-conductive pathways. Thus, the thermal conductivity increased remarkably at high filler concentration.

### Electrical properties analysis

Insulating materials usually work in the environment with high current intensity and electrical field intensity. To ensure the electrical equipment operates fluently, the eligible electrical properties including dielectric polarization, dielectric loss, electrical insulation and electrical breakdown were necessary for insulating materials.

**Fig 5:**
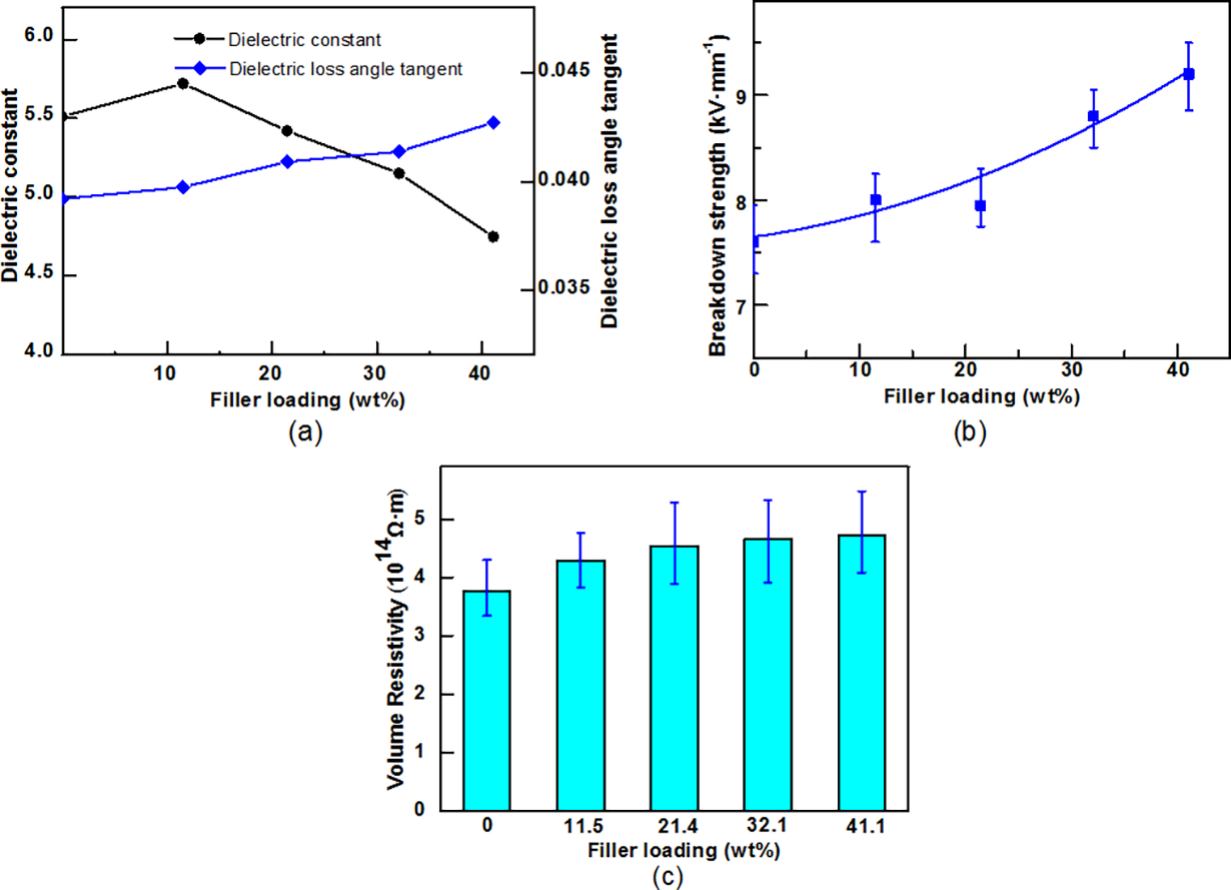
The effects of filler loading on (a) Relative permittivity and dielectric loss angle tangent (b) breakdown strength (c) Volume resistivity.

In the electrical field, the positive and negative charges of the dielectric molecular will transfer towards two opposite direction of poles to polarize the dielectric molecular on the surface. The relative dielectric constant, abbreviates to ε_γ_, is a physical quantity to quantify the degree of polarization of the dielectric. Fig 5(A) shown the ε_γ_ of the composites. The main ingredient of the resultant composites was fibre, which has abundant polar hydroxyl groups on each structural unit of the cellulose molecules. The ε_γ_ of pure cellulose molecule was approximately 6.5 [31]. However, the composites possessed a network-like porous structure, and these micropores were filled up with the air which has a ultralow ε_γ_ of about 1. Hence the ε_γ_ of the material actually measured is much less than the ε_γ_ of pure cellulosic molecule [32]. The sample with no BN filler addition had a ε_γ_ of 5.51. With the filler loading of 11.51 wt%, the ε_γ_ increases to a maximum of 5.72. Then the ε_γ_ decreases when the filler loading continued to increase. A small amount of positively charged groups were introduced into the composites, such as the retention aids of cationic polyacrylamide and the amino groups from the grafted APTES. These polar molecules were susceptible to polarization in the electrical field, and resulted in a certain increase of ε_γ_. As the filler loading increased, owing to the constant grammage of the composites, it can be considered that a part of cellulosic fibres were replaced by h-BN fillers (ε_γ_ of about 4), which possessed a better dielectric property compared with cellulosic molecule. As a consequence, the ε_γ_ of the composites were reduced.

The polarization and conductance of dielectric materials always accompany with energy loss [3]. The dielectric dissipation factor, so called the tangent of dielectric loss angle (tgδ), is a parameter to reflect the degree of energy loss inside the dielectric. As Fig 5(A) shown, the tgδ of the BN/cellulosic fibre composite increased slightly with the increased filler content. However, it still was kept in the excellent low range [6].

In high voltage electrical equipment, the breakdown strength is a significant performance parameter for insulating materials. Fig 5(B) reflected that the effects of BN fillers on the breakdown strength of the composites. The enhancement arose for two main reasons. First, as mentioned above, the h-BN filler possessed a relatively similar dielectric constant with cellulose, such a tiny difference could effectively restrain the electric field distortion [18]. Second, h-BN fillers gradually occupied the intervals within the composites. The plane BN particles might restrain the movement of the polar groups. Thus, the resultant composites exhibited higher breakdown strength.

Fig 5(C) showed the volume resistivity of the composites. The volume resistivity of the resultant composites reached 4.72×10^14^ Ω•cm, increased by 27% compared with the pure fibres samples. The composites exhibited a higher volume resistivity with h-BN fillers concentration. As the filler loading increased to approximately 20wt%, the volume resistivity increased gradually and steadily. The reason was probably that the filler particles could aggregate at a high filler loading and cannot uniformly disperse within the composites, so that the BN fillers made less contribution to the volume resistivity.

### Morphology analysis

**Fig 6:**
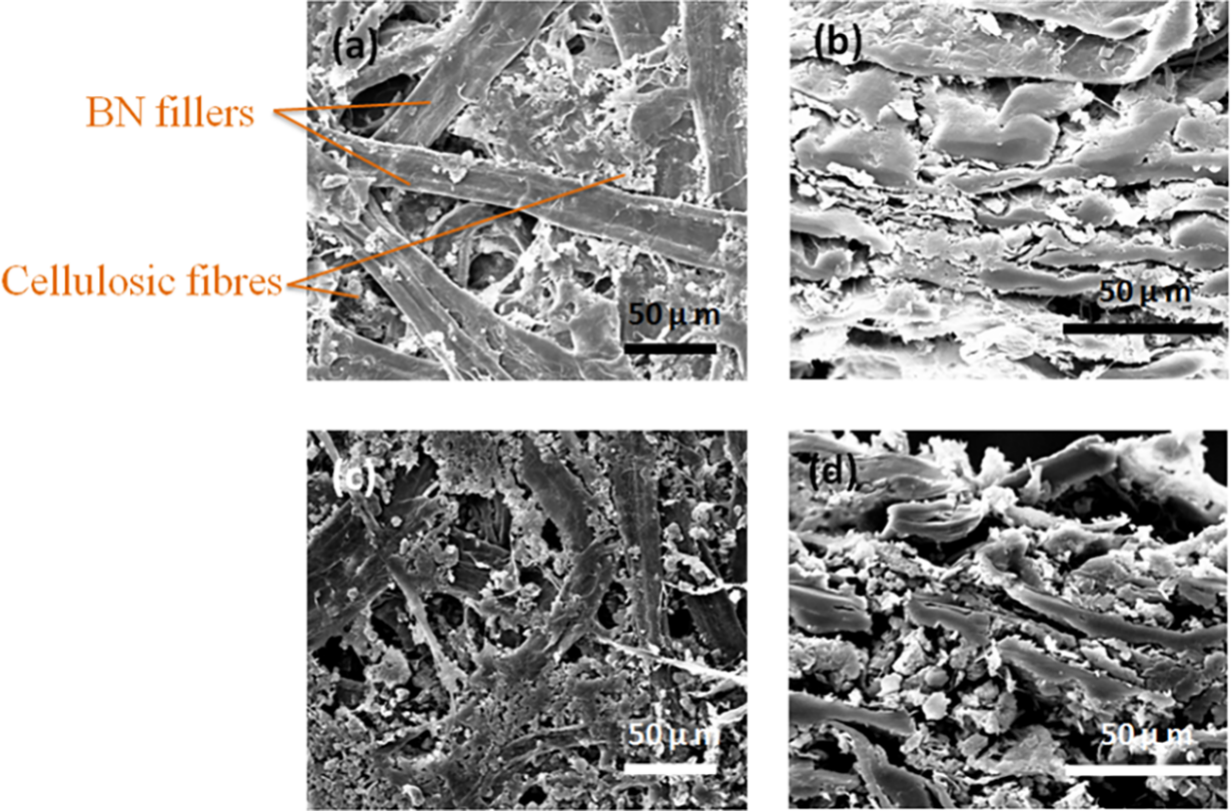
SEM images of (a) surface and (b) cross-section of composite with 11.5wt% filler loading; SEM images of (c) surface and (d) cross-section of composite with 41.1wt% filler loading.

The surface and cross-sectional SEM images of the BN/cellulosic fibre composite were shown in Fig 6A–6D. There were micron-sized voids between the cellulosic fibre matrix, and the plane BN filler particles dispersed in the intervals. At a low filler loading, the BN particles were more evenly introduced onto the cellulosic surface scatteredly. The BN particles kept a certain distance with each other and contact rarely. However, when at a high filler loading, it can be observed that the voids gradually occupied by BN particles. These particles contact one by one with each other along the cellulosic fibre skeleton. In this case, the increased contact area between the h-BN filler particles formed network within the composite, which was the so-called heat-conductive pathway. And this kind of pathways was just the desirable structure that can reduce the thermal resistance of the composite.

## Conclusion

Herein, the effects of BN loading on the BN/cellulosic fibre insulating materials were evaluated. With the increase of BN fillers loading, the BN particles gradually contacted with each other to form chain-like or network-like structure which called heat-conductive pathways. The composites exhibited higher thermal conductivity and better electrically insulation properties. Grafting APTES and dual-sized fillers significantly enhanced the thermal conductivity of the composites. As a result, the thermal conductivity of the composites with dual-sized surface modified BN fillers enhanced by 387% at a h-BN loading of 41.08 wt%. In brief, the BN/cellulosic fibre composites possessed application potential in high voltage insulation field.
